# Outlier detection using iterative adaptive mini-minimum spanning tree generation with applications on medical data

**DOI:** 10.3389/fphys.2023.1233341

**Published:** 2023-10-13

**Authors:** Jia Li, Jiangwei Li, Chenxu Wang, Fons J. Verbeek, Tanja Schultz, Hui Liu

**Affiliations:** ^1^ School of Software Engineering, Xi’an Jiaotong University, Xi’an, China; ^2^ Leiden Institute of Advanced Computer Science, Leiden University, Leiden, Netherlands; ^3^ Department of Geriatric Surgery, The Second Affiliated Hospital of Xi’an Jiaotong University, Xi’an, China; ^4^ MOE Key Lab of Intelligent Network and Network Security, Xi’an Jiaotong University, Xi’an, China; ^5^ Cognitive Systems Lab, University of Bremen, Bremen, Germany

**Keywords:** minimum spanning tree, outlier detection, cluster-based outlier detection, data mining, medical data

## Abstract

As an important technique for data pre-processing, outlier detection plays a crucial role in various real applications and has gained substantial attention, especially in medical fields. Despite the importance of outlier detection, many existing methods are vulnerable to the distribution of outliers and require prior knowledge, such as the outlier proportion. To address this problem to some extent, this article proposes an adaptive mini-minimum spanning tree-based outlier detection (MMOD) method, which utilizes a novel distance measure by scaling the Euclidean distance. For datasets containing different densities and taking on different shapes, our method can identify outliers without prior knowledge of outlier percentages. The results on both real-world medical data corpora and intuitive synthetic datasets demonstrate the effectiveness of the proposed method compared to state-of-the-art methods.

## 1 Introduction

Massive and complex databases often contain numerous patterns. Most traditional data mining tasks find general patterns in the datasets and regard the outliers as noise, such as frequent pattern mining, classification, and clustering. What should not be overlooked is that outliers may embody more valuable information than general patterns, as they could imply abnormal behaviors or potential new patterns, which is consistent with real-life situations Liu and Schultz (2022). An outlier generally means a point that deviates greatly from others, typically generated by a different mechanism Atkinson and Hawkins (1980). Detecting outliers in a dataset is critical and beneficial for practical applications in various fields, such as fraud detection Fiore et al. (2019); Tseng et al. (2015), cyber-security, medical diagnostics Schlegl et al. (2017); Zhang et al. (2016), and others Kang et al. (2016). Outliers of physiological signals in the form of time series are often studied by statistical models, with the latest examples including self-similarity matrices Rodrigues et al. (2022) and subsequence search Folgado et al. (2022), while graph theory-based outlier detection algorithms shine in medical data composed of discrete points, the subject of this article.

Research on outlier detection has a long tradition. Following Hawkins’ classical definition of outliers Atkinson and Hawkins (1980), researchers have developed various outlier detection algorithms and schemes over the years. Generally speaking, these approaches fall into four major groups: distribution-based Zong et al. (2018), distance-based Amagata et al. (2021); Radovanović et al. (2015), density-based Schubert et al. (2014); Corain et al. (2021), and clustering-based Manzoor et al. (2016); Chawla and Gionis (2013); Wang et al. (2019). The main characteristic of the distribution-based method is that it fits datasets with a standard distribution, assuming that the underlying distribution of the dataset is known in advance. It identifies the outliers as the points that do not conform to a particular distribution that sums up most of the data points. Although effective for datasets with a known distribution, the distribution-based approach is not always advisable for real-world scenarios due to the unavailability of *a priori* distribution knowledge and the high cost of concluding an appropriate distribution Li et al. (2022). During the past 2 decades, distance-based methods have attracted much attention, finding points whose given distance range of neighbors contains less than a predetermined percentage of points of the whole dataset Knorr and Ng (1998). In addition to the unavoidable computational expense of the distances between all pairs, the configuration of the neighboring amount *k* significantly influences the detection quality. The density-based algorithm was proposed to cover the shortcoming of distance-based approaches, which often fail to detect local outliers. The local outlier factor (LOF) proposed by Markus is widely used to evaluate the outsiderness degree of a point Jahanbegloo and Jahanbegloo (2000), performing well in the dataset with different density distributions. LOF measures the difference between the samples’ local density and their *k*-nearest neighbors (*k*-NN) as the outlier factor. However, the choice of *k* can greatly influence performance. Clustering-based methods have gained popularity in the field of outlier detection as they can overcome the influence of parameters. Clustering divides the dataset into several clusters, making the intra-cluster distance much smaller than the inter-cluster distance. Outliers are identified as the points that are isolated from the resulted clusters. Many researchers have focused on combining clustering and outlier detection Wang et al. (2019); Degirmenci and Karal (2022); Liu et al. (2019). Clustering based on minimum spanning trees (MSTs) is widely adopted for its ability to identify clusters with irregular boundaries Wang et al. (2013). Unlike *k*-means, there is no assumption that the data points are grouped around centers or separated by a regular geometric curve. However, building an MST is time-consuming for large datasets and may not detect different density clusters effectively Li et al. (2019).

This article proposes a novel outlier detection method, called Mini-MST-based Outlier Detection (MMOD), which does not require specifying the number of outliers. For the emerging real-world data without ground truth, sometimes called black-box data, algorithms that do not require a predetermined number or proportion of outliers can often be straightforwardly plug-and-play. Our approach uses a new distance measure as the edge weight of MST, to better differentiate the clusters so that the outliers in datasets with various density clusters can be identified. To improve the efficiency, we compute some mini-MSTs with a small proportion of the whole dataset and delete the points added to the trees. Our method starts with constructing a Prim’s MST to find one data point in the densest cluster. Subsequently, some small mini-MSTs are computed from the densest point using a distance scaled by the termination threshold of Prim’s algorithm instead of the traditional Euclidean distance to represent the edge weight. The points in each mini-MST can be regarded as a cluster. We compute a termination condition for the MST construction so that the remaining points are outliers after all the mini-MSTs are constructed. The novelty of the proposed method includes a new distance measure to construct the MST to identify different density clusters and efficiency enhancement by employing the mini-MST structure and deleting the data points while constructing the trees. Compared with eight state-of-the-art outlier detection methods on various real-world medical datasets and five synthetic datasets, our method’s feasibility and effectiveness will be proven.

The remainder of the article is organized as follows. Section 2 discusses relevant work on outlier detection. Section 3 prepares the foundations of the preliminaries and definitions for subsequent tasks. Section 4 presents our mini-MST-based outlier detection method. Section 5 manifests the experimental results in comparison to the state-of-the-art technologies. Section 6 concludes our work and looks into the future.

## 2 Related work

### 2.1 Distance-based outlier detection

Knorr and Ng advocated distance-based outlier detection (DOD) for the first time to soften the limitation of distribution-based methods on data distribution and prior information Knox and Ng (1998). The local distance-based outlier factor (LDOF) is one of the most known variants in distance-based approaches Zhang et al. (2009), which measures the outsiderness degree in scattered real-world datasets. The relative location of one point and its neighbors evaluates the deviation of the patterns, based on which the classical top-*n* strategy chooses outlier candidates. As the volume of data increases and the form of data diversifies, data streams are becoming popular, spawning many studies on in-stream outlier detection. Angiulli et al. presented three algorithms to detect distance-based outliers in a sliding-window model Angiulli and Fassetti (2010). A novel notion called the one-time outlier query identifies outliers in a targeted window at an arbitrary time. Milos Radovanovic et al., focusing on the effects of high-dimensional datasets, analyzed the relationship between antihubs and outliers taking into account the reverse nearest neighbor, that is, the point neighboring its *k*-NN Radovanović et al. (2015). Continuous outlier mining employs the sliding-window data structure to reduce time and memory costs, which is flexible in terms of input parameters Kontaki et al. (2016). Scaleable, distributed algorithms have been put forward for substantial data. MapReduce works for distributed tasks: A multi-tactic strategy for DOD is proposed, where data characteristics are considered in data partitioning Cao et al. (2017). The in-memory proximity graph copes with the memory problem of large datasets, analyzing the type of proximity graph for the algorithm Amagata et al. (2022).

### 2.2 Minimum spanning tree-based outlier detection

MST is an important and widely used data structure in clustering analysis. MST-based clustering finds inconsistent edges and deletes them to form reasonable and meaningful clusters. In the case of the existence of outliers, cutting inconsistent edges can result in isolated points or clusters, which can be utilized for outlier detection.

Jiang et al. proposed a two-phase outlier detection method based on *k*-means and MST, in which small clusters are selected and deemed outliers Jiang et al. (2001). There are two stages to this method. In the first phase, they used modified *k*-means clustering by assigning the far point as a new cluster center. In the second phase, an MST is constructed, and the longest edges are cut to find the small clusters, the tree with a few nodes. MST-based spatial outlier detection combines MST-based clustering constructed by the Delaunay triangle irregular net (D-TIN) and density-based outlier detection, performing effectively on the data of soil chemical elements Lin et al. (2008). Previous work also modified the *k*-means algorithm to construct a spanning tree efficiently Wang et al. (2012). Integrating MST-based clustering and density-based outlier detection improves the quality of detection. Meanwhile, the removal of outliers may lead to enhanced results of MST-based clustering Wang et al. (2013).

### 2.3 Summary of deficiencies

From the existing work in outlier detection, it can be concluded that.

• Distance-based models are weak in detecting local outliers. Furthermore, the boundary points in a sparse cluster may be misclassified as outliers.
• Density-based models are less effective at identifying global outliers because these outliers are usually scored low.
• Clustering-based models, ignoring the locations and conditions, can identify outliers that do not belong to any cluster, but are not robust to the presence of different density clusters.

We propose a novel method inspired by MST to tackle the shortcomings mentioned above.

## 3 Foundation

### 3.1 Preliminaries

Spanning tree. Given *N n*-dimensional data points (vertices) in Euclidean space, the spanning tree is a tree that includes all *N* vertices without closed loops, in which the number of edges is not greater than $\frac{N\left(N-1\right)}{2}$ because full connectivity is not required.

Minimum spanning tree (MST). An MST is a spanning tree whose total weight is minimal among all spanning trees, which means that the number of edges in an MST is *N* − 1. The total weight is the sum of the weight of all edges of the tree. Generally speaking, the weight of an edge in a tree is the Euclidean distance between its two endpoints. Mahalanobis distance or other metrics can also be used as a measure.

Prim’s MST. Among the three traditional algorithms for constructing MST, Prim, Kruskal, and Boruvka, this work employs Prim Medak (2018), whose process can be briefly described as.

• Randomly choose one point in the dataset as the root of the tree;
• Compute the pairwise distances between the chosen point and other points to find the shortest edge;
• Add the shortest edge and the other endpoint of it to the tree;
• Repeat the steps above until all the data points are added to the tree.

Euclidean distance (*d*). Given two endpoints *x*_1_ and *x*_2_ of the *ith* edge *e*_*i*_ of an MST in the *n*-dimensional Euclidean space, the Euclidean distance between *x*_1_ and *x*_2_ is

$$d_{i}=d\left(x_{1},x_{2}\right)=\sqrt{\overset{n}{\underset{j=1}{\sum }}{\left(x_{1j}-x_{2j}\right)}^{2}}.$$ (1)

### 3.2 Definitions

Threshold of termination (*T*_*t*_). A global termination threshold sets the stopping condition of the cluster computation to identify the remaining points as outliers, defined as

$$T_{t}=\bar{d}+\sqrt{\overset{N-1}{\underset{i=1}{\sum }}{\left(d_{i}-\bar{d}\right)}^{2}},$$ (2)

where $\bar{d}$ is the average weight of all edges {*e*_*i*_} in the Prim’s MST constructed of the dataset, computed as

$$\bar{d}=\frac{\sum_{i=1}^{N-1}d_{i}}{N-1},$$ (3)

where the numerator accumulates all edges in the Prim’s MST.

Threshold-based Euclidean distance (*ted*). We put forward a weighted Euclidean distance to replace the traditional Euclidean, computed as

$$ted\left(x_{1},x_{2}\right)=\frac{d\left(x_{1},x_{2}\right)}{T_{t}}.$$ (4)

*T*_*t*_ is calculated based on all edges from the MST of the entire dataset, which enables the scaled distances to handle different density clusters by reducing the discrepancy of the edge weights.

Mini-MST generation. In this work, the mini-MST generation algorithm starts from a point in the densest cluster and computes the MST using *ted*. When an edge is supposed to be added to the tree, its weight is first compared to the adaptive exit condition defined below. If the former is greater, the other end of the current edge does not belong to the current cluster. Consequently, the computation of the current mini-MST terminates and a new construction starts.

Mini-edge weight set (*MEW*). A mini-edge weight set records the weight of the edges added to the mini-MST. Once an edge is added to the MST, its weight enters *MEW*.

The first value added to *MEW*, denoted as *MEW*_1_, defaults to *d*_1_, the length of the first edge added to mini-MST. The default value performs well on all real-world datasets applied in this work, as Section 5.2 manifests. In exceptional cases, like a significantly high value of *d*_1_, *MEW*_1_ can be tuned, such as for the synthetic “Two densities” and “Three clusters” datasets in Appendix, where *MEW*_1_ was set to 1.

Adaptive exit condition of mini-MST generation (aec). To improve efficiency, we repeatedly compute mini-MSTs and delete the points added to the MST, applying an adaptively updated exit condition that judges whether the mini-MST generation should terminate at the targeted edge *e*_*i*_:

$$aec\left(e_{i}\right)=\bar{MEW}+\sqrt{\overset{\left|MEW\right|}{\underset{i=1}{\sum }}{\left(MEW_{i}-\bar{MEW}\right)}^{2}},$$ (5)

where $\left|MEW\right|$ and $\bar{MEW}$ are the size and the average weight of the current *MEW* that *e*_*i*_ is supposed to enter, respectively.

MST-based outliers. MST-based outliers are the points not added to any generated mini-MSTs. Our method does not require a given number of outliers; Instead, *aec* and *T*_*t*_ differentiate the different density clusters and outliers. The construction of the mini-MSTs finishes when the weight of the next edge is greater than the threshold, so that the points in this current mini-MST can be regarded as a cluster with the same density. Furthermore, a sliding window is applied to the edge weight denoted by the Euclidean distance. If the mean value of such a window is greater than *T*_*t*_, the remaining points that are not ready to be added to the tree should be deemed outliers.

## 4 Methods

### 4.1 MST generation details and an illustrative example

In response to traditional MST-clustering-based outlier detection’s weak performance on datasets with different densities, this work applies a novel distance measure scaled by the threshold of algorithm termination for better discrimination of normal points and outliers. Such a threshold could be considered a quasi-measure of noise in the dataset. The second algorithm improvement of this work targets efficiency: Mini-MSTs are built iteratively. Finishing a mini-MST generation in a cluster is followed by the deletion of processed points and a new construction procedure on the remaining points. An adaptive exit condition based on a progressively updated *MEW* qualifies the termination of the mini-MST building. A traditional MST algorithm, like Prim, is first applied to create an exact MST to find the point in the densest cluster. Subsequently, all edges are sorted in non-decreasing order to ensure that the first edge’s two endpoints are in the densest cluster because the higher the cluster’s density, the shorter the distances between its points. The edges between different density clusters are taken into account.

**FIGURE 1:**
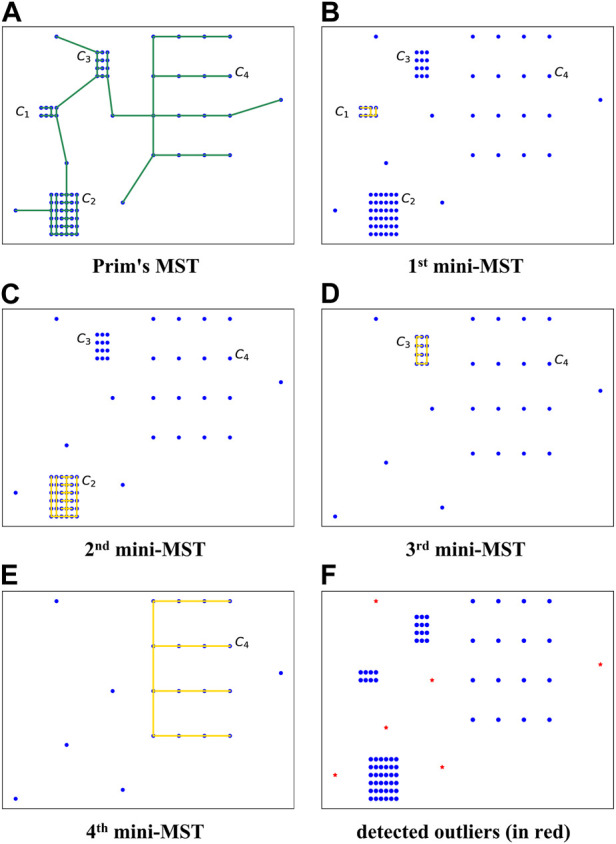
An intuitive example of the adaptive mini-minimum spanning tree-based outlier detection (MMOD) method. *C*_1_, *C*_2_, *C*_3_, and *C*_4_: four clusters of different densities; **(A)** Prim’s MST on the original dataset; **(B)**–**(E)** procedure of iterative mini-MST construction; **(F)** detected outliers (in red).

Figure 1 illustrates a simplified case that embodies four clusters with different densities and six outliers. *C*_1_ is the densest cluster with the smallest average weight of edges. A Prim’s MST is constructed first to find the point in the densest cluster, as Figure 1A demonstrates. The shortest edge can be identified by sorting the edges in Prim’s MST in non-decreasing order. Let *s* denote the start point of the shortest edge in *C*_1_, from which a mini-MST is computed. Like Prim, the shortest edge is repeatedly added to the mini-MST until the next edge’s weight is larger than the exit condition *aec* (see Eq. (5)). The points in the built mini-MST are labeled normal and removed from the dataset. The above steps are repeated from the point of the next densest cluster, which in this example is *C*_2_, and the whole procedure ends with the adaptive exit condition being satisfied. The remaining points are considered outliers.

### 4.2 Adaptive mini-minimum spanning tree-based outlier detection (MMOD)

As can be observed from the example in Section 4.1, the proposed method is centered on the iterative computation of mini-MSTs. Similarly to Prim, two arrays, *labeled*_*data* and *unlabeled*_*data*, are used to record data points added or not added to the tree, initialized by an empty set and all points, respectively. Unlike the traditional exact MST, the MST in our algorithm is constructed according to the data density, and an exit condition is added to obtain a mini-MST for efficiency. The edge weight of the mini-MST is a threshold-based Euclidean distance in place of the conventional Euclidean distance (see Eq. 4). *s* denotes the start point of the mini-MST. Aside from the MST array used in Prim’s MST, an additional *ted*_*arr* records the threshold-based distance between all data points. Algorithm 1 details the mini-MST construction.

Algorithm 1 Mini-minimum spanning tree construction

**Require:** a set of *N* data points, *R*; start point, *s*; *labeled*_*data*; *unlabeled*_*data*
**Ensure:** an MST
1: Let ***MEW*** denote mini edge weight set
2: Let ***result_set*** denote the generated MST
3: Let ***ted_arr*** denote *N* − 1 threshold-based Euclidean distances
4: Let ***edge_arr*** denote the parents of the *N* data points
5: **for** i ← 1: N **do**
6:  ***edge_arr*** [*i*] ← *s*
7: $ted\text{\_}arr\left[i\right]\leftarrow ted\left(s\right.,i_{th}$ point in *R*) (see Equation 4)
8: **end** **for**
9: choose another point *p* from *R* which is the nearest point to *s*
10: add the edge denoted by *s*, *p*, ***ted_arr*** [*s*] to ***result_set***
11: move *p* from *unlabeled*_*data* to *labeled*_*data*
12: initialize *MEW* with distance (*s*, *p*)
13: **while** True **do**
14:  initialize min _*ted* with *∞*;
15:  **for** *q* in *unlabeled*_*data* **do**
16:   *last*_*weight* ← *ted* (*p*, *q*)
17:   **if** *last*_*weight* < ***ted_arr*** [*q*] **then**
18:    update ***ted_arr*** with the *last*_*weight*
19:    update ***edge_arr*** with the index of *p*
20:    min _*ted* ← *last*_*weight*
21:   **end** **if**
22:  **end** **for**
23:  compute *aec* (*e*_*i*_) according to Equation 5
24:  **if** min _*ted* > *aec* (*e*_*i*_) **then** break
25:  **end** **if**
26:  choose point *r* with smallest ted in ***ted_arr***
27:  add the edge denoted by *p*, *r*, ***ted_arr*** [*p*] to ***result_set***
28:  move *r* from *unlabeled*_*data* to *labeled*_*data*
29:  update *p* with *r*
30:  add ***ted_arr*** [*p*] to *MEW*
31: **end** **while**
32: **return** ***result_set***, ***edge_arr*** and ***ted_arr***

Least number. To be noted, the number of points in the cluster falls within a certain range. A cluster containing too few points is considered an outlier cluster. *least*_*number* distinguishes normal clusters from outlier clusters:

$$least\text{\_}number=ROUND\left(\sqrt{\frac{N}{n}}\right),$$ (6)

where the *ROUND* function finds the closest integer to the parameter; *N* and *n* are the size and dimension of the dataset, respectively. If the number of edges of a mini-MST is less than *least*_*number*, the points belonging to the tree are marked as outliers.

Since the algorithm starts building MSTs from the densest cluster, it keeps outliers until all mini-MSTs have been generated. Therefore, the threshold of termination *T*_*t*_ (see Eq. 2) can be applied to stop finding normal points. To compare with *T*_*t*_, a window filled with the weights of the current edge and the following five edges is used: If the mean value of this window is greater than *T*_*t*_ (see Eq. 2), the remaining data points will be treated as outliers.

Algorithm 2 provides the pseudocode of the proposed adaptive mini-MST-based outlier detection, which takes the input of the dataset *R* with *N* data points and its corresponding Prim’s MST denoted by the edges. Each edge of the MST consists of a starting point, an endpoint, and an edge weight.

Algorithm 2 Adaptive mini-minimum spanning tree-based outlier detection

**Require:** dataset *R*
**Ensure:** a label array, ***labels***
1: ***labels*** ← [−1]*N
2: compute a Prim’s MST using Prim algorithm
3: sort the Prim’s MST in non-decreasing order
4: compute *T*_*t*_ according to Equation 2
5: compute the *least*_*number* according to Equation 6
6: **for** edge in MST **do**
7:  *s* ←start point of edge
8:  **if** one of the two ends of the edge is in *labeled*_*data* **then**
9:   continue
10:  **end** **if**
11:  *window* ← the weight of the current edge and the next 5 edges;
12:  *edge*_*threshold* ← the mean value of *window*
13:  **if** *edge*_*threshold* < *T*_*t*_ **then** *mini*_*mst* ← *Mini*_*MST* (*DS*, *s*, *labeled*_*data*, *unlabeled*_*data*)
14:   **if** len (*mini*_*mst*) >least_number **then**
15:    labeled the two ends of the edges in *mini*_*mst* as normal
16:   **end** **if**
17:  **else**
18:   break
19:  **end** **if**
20: **end** **for**
21: **return** ***labels***

## 5 Experimental results and evaluation

### 5.1 Applied datasets

Ten experiments were conducted on different real-world datasets, as summarized in Table 1, to demonstrate MMOD’s applicability on the benchmark Campos et al. (2016). The datasets will be introduced in detail in Section 5.4, along with the results of the experiments carried out on them.

**TABLE 1:** Description of the applied real-world datasets.

Dataset	Number of Samples	Number of Outliers	Number of Attributes
HeartDisease	270	120	13
Parkinson	195	147	22
Pima	768	268	8
SpamBase	4601	1813	57
WDBC_v05	367	10	30
WDBC_v06	367	10	30
WDBC_v07	367	10	30
WDBC_v08	367	10	30
WDBC_v09	367	10	30
WDBC_v10	367	10	30

As a supplement, experiments on five synthetic two-dimensional datasets with different morphologies are added to demonstrate MMOD’s parameter tuning and its availability on manually generated data; plus, the two-dimensional visualization is intuitive and well-readable (see Appendix).

### 5.2 State-of-the-art methods for comparison

MMOD’s experimental results were compared with eight algorithms from the Python outlier detection package Zhao et al. (2019), including four classical algorithms, *k*-NN Ramaswamy et al. (2000), LOF Jahanbegloo and Jahanbegloo (2000), angle-based outlier detection (ABOD) Pham and Pagh (2012), and histogram-based outlier score (HBOS) Goldstein and Dengel (2012), as well as four recent algorithms, one class support vector machine (OCSVM) Erfani et al. (2016), lightweight online detector of anomalies (LODA) Pevný (2016), locally selective combination of parallel outlier ensembles (LSCP), and multiple-objective generative adversarial active learning (MOGAAL) Liu et al. (2019) Zhao et al. (2018). LOF and *k*-NN are classical density-based and distance-based methods, respectively. ABOD is developed for high-dimensional feature space datasets to alleviate the “curse of dimensionality,” an efficient version of which was used in our experiments. HBOS is an unsupervised outlier detection method that computes the outsiderness degree by building histograms. OCSVM is an extension of the support vector algorithm that learns a kernel function called the decision boundary, distinguishing outliers from inliers. LODA is operative for data streams and real-time applications. LSCP, also unsupervised, chooses the competent detectors by using the local region of the data points. The newly presented MOGAAL is based on a generative adversarial active learning neural network.

To generate a fair comparison reference, the *k*_*threshold*_ value for each state-of-the-art method being compared was set to 7, a typical value setting. Literature such as Campos et al. (2016) records the performance of other *k*_*threshold*_ values on most reference methods. The outlier percentage is calculated as the number of outliers divided by the size of the dataset. All experiments were run through Python 3.6.5 on a computer with an Intel^®^ Core™ 3.2 GHz i5-3470 CPU and 4 GB RAM.

### 5.3 Evaluation metrics

Conventional evaluation metrics precision, recall, and *F*-measure were applied to analyze and compare the experimental results on real-world datasets. Let *m* denote the number of correct outliers returned by the detector, *n* denote the total number of all outliers returned by the detector, and *o* denote the number of ground-truth outliers. The precision *P* is the proportion of correct outliers in all outliers identified by the detector:

$$P=\frac{m}{n}.$$ (7)

The recall *R* is the proportion of correct outliers that the detector returns in all ground-truth outliers:

$$R=\frac{m}{o}.$$ (8)

The *F*-measure is the harmonic mean of precision and recall:

$$F=\frac{2}{\frac{1}{P}+\frac{1}{R}}=\frac{2PR}{P+R}.$$ (9)

### 5.4 Results on real-world datasets

**FIGURE 2:**
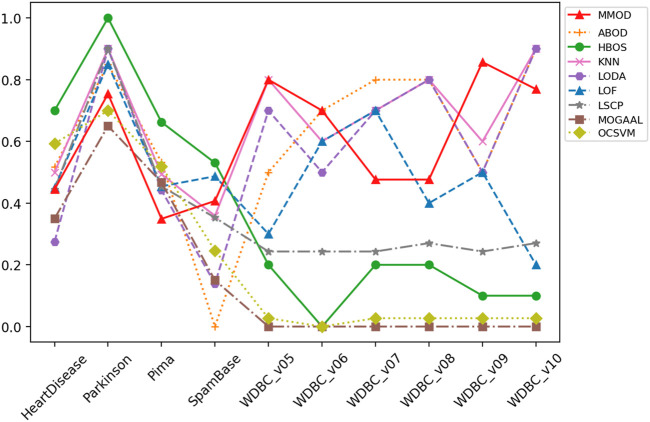
The precision of MMOD’s and peer methods’ experimental results on the real-world datasets.

**FIGURE 3:**
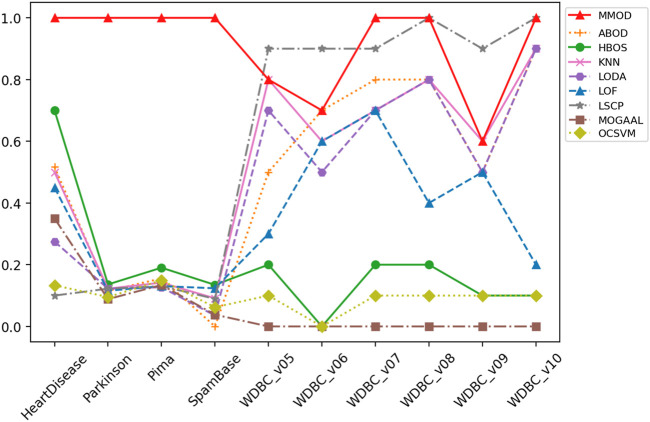
The recall of MMOD’s and peer methods’ experimental results on the real-world datasets.

**FIGURE 4:**
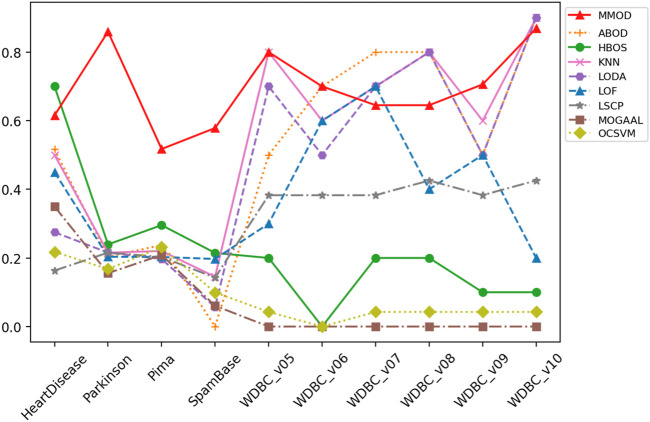
The *F*-measure of MMOD’s and peer methods’ experimental results on the real-world datasets.

Applying real-world datasets can demonstrate the effectiveness of the proposed method straightforwardly. Since medical data are one of the most prominent application scenarios of outlier detection, nine widely investigated open-source medical datasets are utilized for experiments. A spam dataset is additionally brought into the experiment as a case for other domain applications. On each real-world dataset, default MMOD parameter settings or formulas defined in Section 3.2 were adopted, such as the *MEW*’s first added value *MEW*_1_, the threshold-based Euclidean distance *ted*, and the exit condition *aec*, which evidences the broad applicability of MMOD without parameter tuning. The precision, recall, and *F*-measure values of MMOD’s and the experimental results of the peer methods’ are entirely recorded in Tables 1–3, of which the statistics are plotted in Figures 2–4 for visual comparison.

**TABLE 2:** The precisions of experimental results from MMOD and eight state-of-the-art algorithms on the real-world datasets. The best performance on each dataset is indicated in bold.

Dataset	MMOD	ABOD	HBOS	KNN	LODA	LOF	LSCP	MOGAAL	OCSVM
HeartDisease	0.44	0.52	**0.70**	0.50	0.28	0.45	0.44	0.35	0.59
Parkinson	0.75	0.85	**1.00**	0.90	0.90	0.85	0.90	0.65	0.70
Pima	0.35	0.53	**0.66**	0.49	0.44	0.45	0.45	0.47	0.52
SpamBase	0.41	0.00	**0.53**	0.36	0.14	0.49	0.35	0.15	0.25
WDBC_v05	**0.80**	0.50	0.20	**0.80**	0.70	0.30	0.24	0.00	0.03
WDBC_v06	**0.70**	**0.70**	0.00	0.60	0.50	0.60	0.24	0.00	0.00
WDBC_v07	0.48	**0.80**	0.20	0.70	0.70	0.70	0.24	0.00	0.03
WDBC_v08	0.48	**0.80**	0.20	**0.80**	**0.80**	0.40	0.27	0.00	0.03
WDBC_v09	**0.86**	0.50	0.10	0.60	0.50	0.50	0.24	0.00	0.03
WDBC_v10	0.77	**0.90**	0.10	**0.90**	**0.90**	0.20	0.27	0.00	0.03

**TABLE 3:** The recalls of experimental results from MMOD and eight state-of-the-art algorithms on the real-world datasets. The best performance on each dataset is indicated in bold.

Dataset	MMOD	ABOD	HBOS	KNN	LODA	LOF	LSCP	MOGAAL	OCSVM
HeartDisease	**1.00**	0.52	0.70	0.50	0.28	0.45	0.10	0.35	0.13
Parkinson	**1.00**	0.12	0.14	0.12	0.12	0.12	0.12	0.09	0.10
Pima	**1.00**	0.15	0.19	0.14	0.13	0.13	0.13	0.13	0.15
SpamBase	**1.00**	0.00	0.13	0.09	0.04	0.12	0.09	0.04	0.06
WDBC_v05	0.80	0.50	0.20	**0.80**	0.70	0.30	**0.90**	0.00	0.10
WDBC_v06	0.70	0.70	0.00	0.60	0.50	0.60	**0.90**	0.00	0.00
WDBC_v07	**1.00**	0.80	0.20	0.70	0.70	0.70	0.90	0.00	0.10
WDBC_v08	**1.00**	0.80	0.20	0.80	0.80	0.40	**1.00**	0.00	0.10
WDBC_v09	0.60	0.50	0.10	0.60	0.50	0.50	**0.90**	0.00	0.10
WDBC_v10	**1.00**	0.90	0.10	0.90	0.90	0.20	**1.00**	0.00	0.10

**TABLE 4:** The *F*-measures of experimental results from MMOD and eight state-of-the-art algorithms on the real-world datasets. The best performance on each dataset is indicated in bold.

Dataset	MMOD	ABOD	HBOS	KNN	LODA	LOF	LSCP	MOGAAL	OCSVM
HeartDisease	0.62	0.52	**0.70**	0.50	0.28	0.45	0.16	0.35	0.22
Parkinson	**0.86**	0.20	0.24	0.22	0.22	0.20	0.22	0.16	0.17
Pima	**0.52**	0.24	0.30	0.22	0.20	0.20	0.20	0.21	0.23
SpamBase	**0.58**	0.00	0.21	0.15	0.06	0.19	0.14	0.06	0.10
WDBC_v05	**0.80**	0.50	0.20	**0.80**	0.70	0.30	0.38	0.00	0.04
WDBC_v06	**0.70**	**0.70**	0.00	0.60	0.50	0.60	0.38	0.00	0.00
WDBC_v07	0.65	**0.80**	0.20	0.70	0.70	0.70	0.38	0.00	0.04
WDBC_v08	0.65	**0.80**	0.20	**0.80**	**0.80**	0.40	0.43	0.00	0.04
WDBC_v09	**0.71**	0.50	0.10	0.60	0.50	0.50	0.38	0.00	0.04
WDBC_v10	0.87	**0.90**	0.10	0.90	0.90	0.20	0.43	0.00	0.04

#### 5.4.1 The HeartDisease dataset

HeartDisease contains 270 instances, of which 120 outliers represent patients, and the rest describe healthy individuals, showing a close number of normal samples and outliers. Normalized, unduplicated data were used for the experiments of the nine algorithms. Overall, all methods did not perform ideally on this dataset. The highest precision and recall were generated by HBOS and MMOD, respectively. Regarding the *F*-measure, MMOD came in second place, slightly below HBOS, while the rest of the methods did not exceed 0.6. It is worth noting that MMOD’s perfect recall. In terms of dataset composition, HeartDisease is the only one from all participating datasets with normal samples and outliers close to half-and-half. With such a high percentage of outliers (only lower than Parkinson), there are only 13 attributes used for detection (the second fewest), which evidences the difficulty of detection. Nevertheless, MMOD detected all outliers without any missing, despite causing many false identifications. In contrast, although HBOS has a higher *F*-measure than MMOD with a 0.80 gap, it has a recall loss of 0.30, which is too high a leakage rate, being insensitive for disease detection.

#### 5.4.2 The Parkinson dataset

To evaluate MMOD’S effectiveness on a large percentage of outliers, we use the normalized, unduplicated Parkinson dataset, consisting of 195 instances, among which 147 are Parkinson’s disease patients as outliers. Due to the outlier percentage being larger than 50%, no parameters were passed to the eight peer algorithms. All methods performed acceptably in terms of precision, but MMOD is the only one standing out in terms of recall, contributing to its far-leading *F*-measure. For comparison, the *F*-measures of all peer algorithms are below 0.5. Compared to HeartDisease, Parkinson has a substantially higher percentage of outliers, over three-quarters, the highest among all datasets applied. Meanwhile, its total number of attributes is sizably greater than HeartDisease, at 22. Regarding Parkinson’s detection, MMOD’s perfect recall means no miss.

#### 5.4.3 The Pima dataset for diabetes

Another normalized, unduplicated medical dataset, Pima, contains 768 cases, including 268 diabetic patients as outliers. Each sample is composed of 8 attributes. MMOD works imperfectly in terms of precision, although all methods are not bright; however, MMOD’s recall and *F*-measure are highlights. Table 1 implies that Pima contains exactly 500 normal samples, which makes the proportion of outliers about 34.90%, roughly one-third of the total data, for which the number of attributes used to describe the samples is the lowest of all the datasets. MMOD succeeded in detecting all diabetic cases but resulted in a certain number of false positives. Similar to Parkinson, on the *F*-measure, which indicates the overall performance, MMOD outperformed the other methods by a large margin, as none of the others exceeded 0.30.

#### 5.4.4 The WDBC corpus and its variation sets for breast cancer

WDBC describes the nuclear characteristics of a breast cancer diagnosis, whose different variation datasets used in our experiments are randomly downsampled from the original classification dataset for outlier detection Zhang et al. (2009). Each variation of WDBC contains 367 samples, among which there are 10 outliers representing malignant cancers, while other instances indicate benign cancers. Therefore, the outlier proportion of the five WDBC datasets is uniform and tiny, about 2.72%, much smaller than others. Nevertheless, a relatively higher number of attributes are used to characterize the samples, reaching 30, the second highest. The nine algorithms, including MMOD, were experimented on the WDBC dataset’s six unnormalized, unduplicated subsets. For all applied WDBC datasets, MOGAAL was unable to identify any outliers, quitting the competition early.

For WDBC_v05, the proposed MMOD achieves the highest precision of 0.8, along with KNN, followed by LODA with 0.7. None of the other methods achieves a precision greater than 0.5 in this dataset. Regarding recall, LSCP achieves 0.9, while MMOD and KNN are 0.8. Nonetheless, LSCP’s *F*-measure is underperforming due to its low precision, while MMOD and KNN win at *F*-measure. MMOD on WDBC_v06 and WDBC_v09 also yielded similar situations of “optimal precision, suboptimal recall, and best *F*-measure,” just that ABOD replaced KNN as the joint winner on WDBC_v06, while MMOD alone performed best on WDBC_v09. It is noteworthy that besides MOGAAL, HBOS and OCSVM also failed on WDBC_v06. MMOD’s performance metrics on WDBC_v07, WDBC_v08, and WDBC_v10 are similar: perfect recalls with non-optimal precisions and *F*-measures.

A perfect recall of 1 means that all true malignancies are found without missing, while suboptimal precision represents the presence of a false positive chance. Overall, MMOD has a relatively high recall on WDBC, slightly inferior to LSCP (MMOD is higher only on WDBC_v06, while on par or lower at rest). Still, given LSCP’s inferior precision, it can be claimed that MMOD works well overall on WDBC_v05–WDBC_v10, as evidenced also by the *F*-measures. It can also be observed from Figure 4 that MMOD’s *F*-measure performance is relatively stable among a group of algorithms.

#### 5.4.5 The SpamBase dataset

Additionally, an email dataset beyond medical scenarios, SpamBase, was applied, which consists of 4,601 objects of 57 attributes, 1,813 of which are spam emails as outliers. It is considerably formidable to detect outliers in such a dataset. Like in WDBC, MOGAAL did not manage to work. MMOD ranks third in precision, while its recall is again far ahead, leading to the winning *F*-measure. In addition to having the most significant number of samples and attributes, SpamBase has a large outlier quantity, accounting for 39.40%. This relatively “big” data witnessed MMOD’s report card of not missing any spam. All other algorithms have weak *F*-measures worse than 0.21.

### 5.5 Comprehensive performance analysis and discussion

MMOD has perfect or nearly perfect recalls on most datasets, which should be attributed to its ability to greatly retain possible outliers, enabled by the adaptive exit condition. Such an adaptive termination mechanism also improves the efficiency of the algorithm. LSCP’s recall performance is comparable to MMOD on WDBS, but on the one hand, its recall is extremely worse than MMOD on the other datasets; on the other hand, its precision on WDBS is also significantly inferior to MMOD. Regarding precision, HBOS works well on four datasets, but is overall unstable and particularly poor on the other six. MMOD is optimal in three datasets and at an average level globally.

Two of the advanced aspects of MMOD are that it does not require the number of outliers as input and that it is outlier quantity and proportion insensitive. Such a characteristic was well reflected in the experimental results. The applied datasets include various outlier percentages, such as a small portion of outliers, a large percentage of outliers, and a close proportion of outliers and normal samples. Evidently, most of the peer methods are affected by such setups. For datasets with a high percentage of outliers, such as HeartDisease, Parkinson, Pima, and SpamBase, HBOS has high precision values; however, for cases with a low percentage of outliers, HBOS’s precision almost hits rock bottom. Worse, its recalls are always poor, no matter the outlier percentage. *k*-NN, LODA, and LSCP are almost the opposite. *k*-NN and LODA’s precision and recall on datasets with a low percentage of outliers are significantly better than the case with a high percentage of outliers. LSCP’s recall is excellent when the percentage of outliers is low; for the high percentage of outliers, LSCP is almost incapable, not to mention its unsatisfying precision all the time. As a comparison, MMOD’s performance is more consistent regardless of the outlier percentage, without dramatically poor metric values. Its recall is especially consistently splendid, its precision is in the middle of the pack, and its *F*-measure is relatively robust, all verifying that MMOD, which does not take outlier numbers or percentages as inputs, works insensitively to outlier quantity and proportion.

Which one of recall and precision is more valued during outlier detection is relevant to the application scenario. For medical data, especially disease diagnosis, recall is related to whether cases with real diseases will be missed. The preliminary validation experiments of MMOD’s method suggest its usability on medical data.

## 6 Conclusion

Outlier detection is an important approach to data mining, which is widely studied in medical scenarios. MST has been widely applied to clustering and outlier detection as an essential data structure in graph theory. In order to overcome the problems in distance-based and density-based outlier detection, an adaptive mini-minimum spanning tree-based outlier detection (MMOD) method was proposed in this article, employing threshold-based Euclidean distances as the edge weight and adaptive exit conditions of mini-MST generation, to improve efficiency. MMOD does not require the outlier percentage as an input parameter, which peer outlier detection algorithms usually need. Moreover, MMOD can detect outliers in datasets with different densities and is insensitive to the outlier proportion and distribution. A series of experiments in real-world medical datasets manifested the promising results of MMOD; additional spam and five synthetic datasets further validated its applicability. Topics on MST-based outlier detection methods, such as the quantitative measurement of outsiderness degree, remain research values in the future.

## Data Availability

The datasets presented in this study can be found in online repositories. The names of the repository/repositories and accession number(s) can be found below: https://github.com/laetella/MMOD.
