# Supplementary material for: Outlier detection using iterative adaptive mini-minimum spanning tree generation with applications on medical data
Source: Front Physiol. 2023 Oct 13;14:1233341. doi: 10.3389/fphys.2023.1233341 (PMC10613083; doi:10.3389/fphys.2023.1233341)
Supplement: Supplementary file 1 [file DataSheet1.pdf]

Appendix to
Outlier Detection Using Iterative Adaptive Mini-Minimum Spanning Tree Generation with Applications on Medical Data

Jia Li [1,2], Jiangwei Li [3] , Chenxu Wang [1,4], Fons J. Verbeek [2,*], Tanja Schultz [5], Hui Liu [5,*]

[1]*School of Software Engineering, Xi'an Jiaotong University, Xi'an, China*
[2]*Leiden Institute of Advanced Computer Science, Leiden University, Leiden, Netherlands*
[3]*Department of Geriatric Surgery, The Second Affiliated Hospital of Xi'an Jiaotong University, Xi'an, China*
[4]*MOE Key Lab of Intelligent Network and Network Security, Xi'an Jiaotong University, Xi'an, China*
[5]*Cognitive Systems Lab, University of Bremen, Bremen, Germany*

Parameter tuning and result visualization on ive synthetic two-dimensional datasets

Five synthetic two-dimensional datasets with different densities, distributions, and outlier numbers, as Table A details, were employed to further validate the effectiveness of the proposed method and to plot more intuitive results of outlier detection. The five synthetic datasets have different morphologies of cluster amount, cluster density, cluster distribution, outlier density, outlier distribution, outlier proportion, and distance between outliers and clusters, which are illustrated in a concise narrative in Table A.

Table A. Description of the five synthetic two-dimensional datasets for further validation and better result visualization. #: Number of.

Synthetic dataset	Morphology	#Samples
"Two densities"	2 clusters of different densities & 2 outliers closer to the dense cluster	98
"Three clusters"	3 clusters & 20 surrounding outliers	441
"Four densities"	4 clusters of different densities & 6 outliers	78
"Uniform outliers"	1 cluster & 50 uniformly distributed outliers	142
"Arbitrary outliers"	2 dense clusters & 100 arbitrarily distributed outliers	304

Unlike real-world data, parameter tuning of the MEW's first added value MEW_1, the threshold of termination T_t, and the exit condition aec was carried out for individual synthetic datasets to achieve reasonable MMOD results. Such a procedure accommodates the effect of the synthetic data's deliberated realization of particular distributions or densities. The tuning for the three parameters is not complicated or exhaustive, where adjusting each parameter is only an either-or option: either the default value/equation expressed in Section 3.2 or another reasonable and unified deformation. For MEW_1, the alternative value

is 1 instead of the length of the first edge added to mini-MST due to a significantly high value of d_1. For T_t and aec, the alternatives omit the standard deviation parts of the original equations and keep the mean terms, which is the most straightforward measure to tune Equations 2 and 5. Table B documents the parameter-tuning results on the five synthetic datasets. The parameter values on various morphological datasets provide a useful reference for future research on other datasets using MMOD.

Table B. Parameter tuning of the five synthetic two-dimensional datasets.

Synthetic dataset	T_t	MEW_1	aec_e
"Two densities"	default	1	default
"Three clusters"	default	1	default
"Four densities"	default	default	$\overline{MEW}$
"Uniform outliers"	$\overline{d}$	default	default
"Arbitrary outliers"	$\overline{d}$	1	default

As illustrated in Figure A, the experimental results on synthetic two-dimensional datasets indicate that the MMOD method is capable of detecting outliers in manually set data with varying morphologies of cluster amount, cluster density, cluster distribution, outlier density, outlier distribution, outlier proportion, and distances between outliers and clusters, given appropriate parameter tuning. The eight state-of-the-art peer methods (see Section 5.2) were also applied to the synthetic datasets for performance comparison. The performance of the nine methods, including MMOD, on the five synthetic datasets is visualized in Figures B–F for reference.

It should be declared again that the above-mentioned parameter tuning in Appendix, although straightforward and efficient, provides only a reference for testing MMOD in the presence of ground truth or for datasets with artificial morphologies and distributions. The experimental results detailed in the main text have verified on various datasets that MMOD produces good results directly without tuning. We do not imply or emphasize tuning the parameters of MMOD for real-world datasets, especially those without ground-truth outlier labels or even black-box cases.

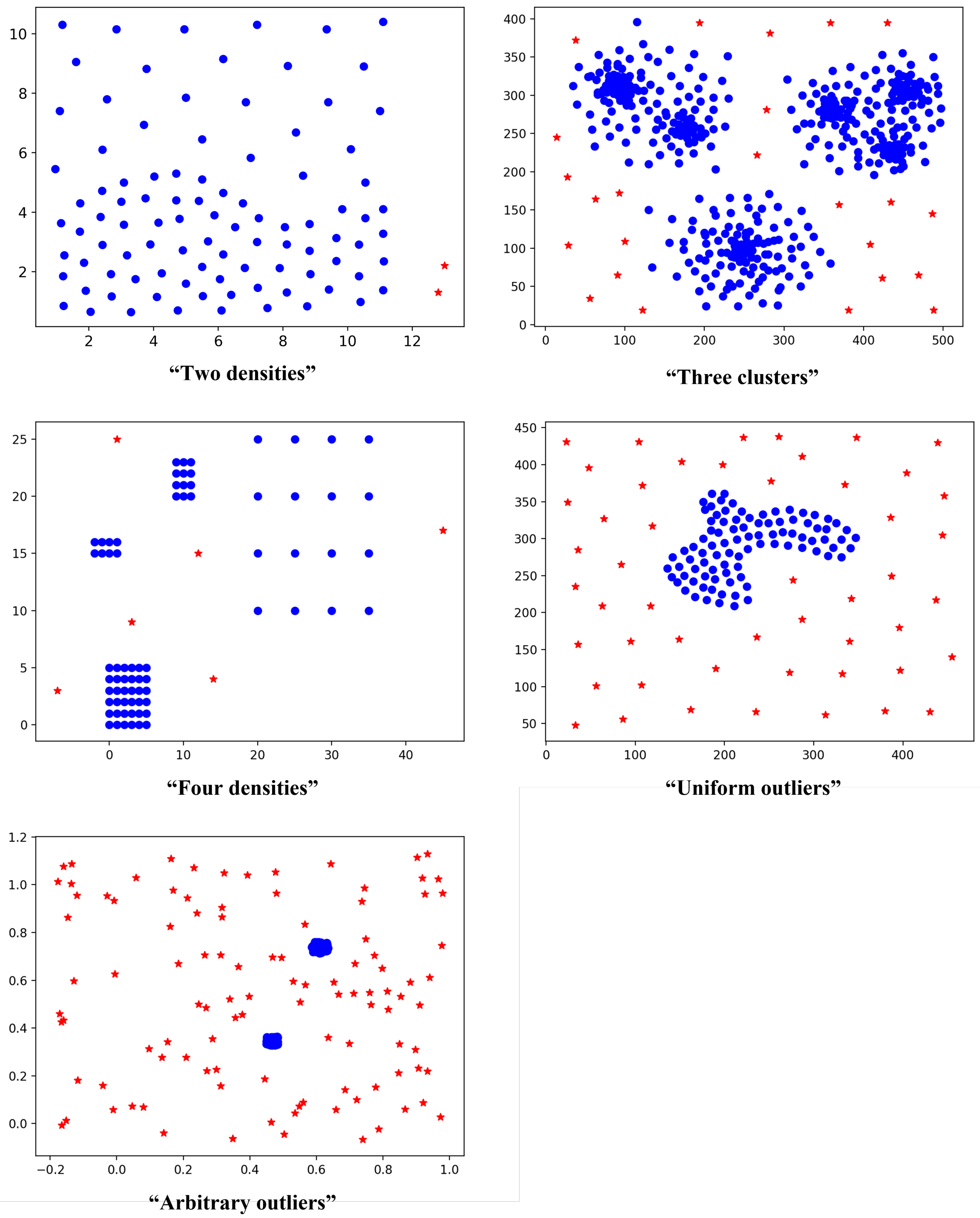

Figure A. Summary of MMOD's outlier detection results on the five synthetic two-dimensional datasets. Red: outliers detected by the proposed adaptive mini-minimum spanning tree-based outlier detection (MMOD) method.

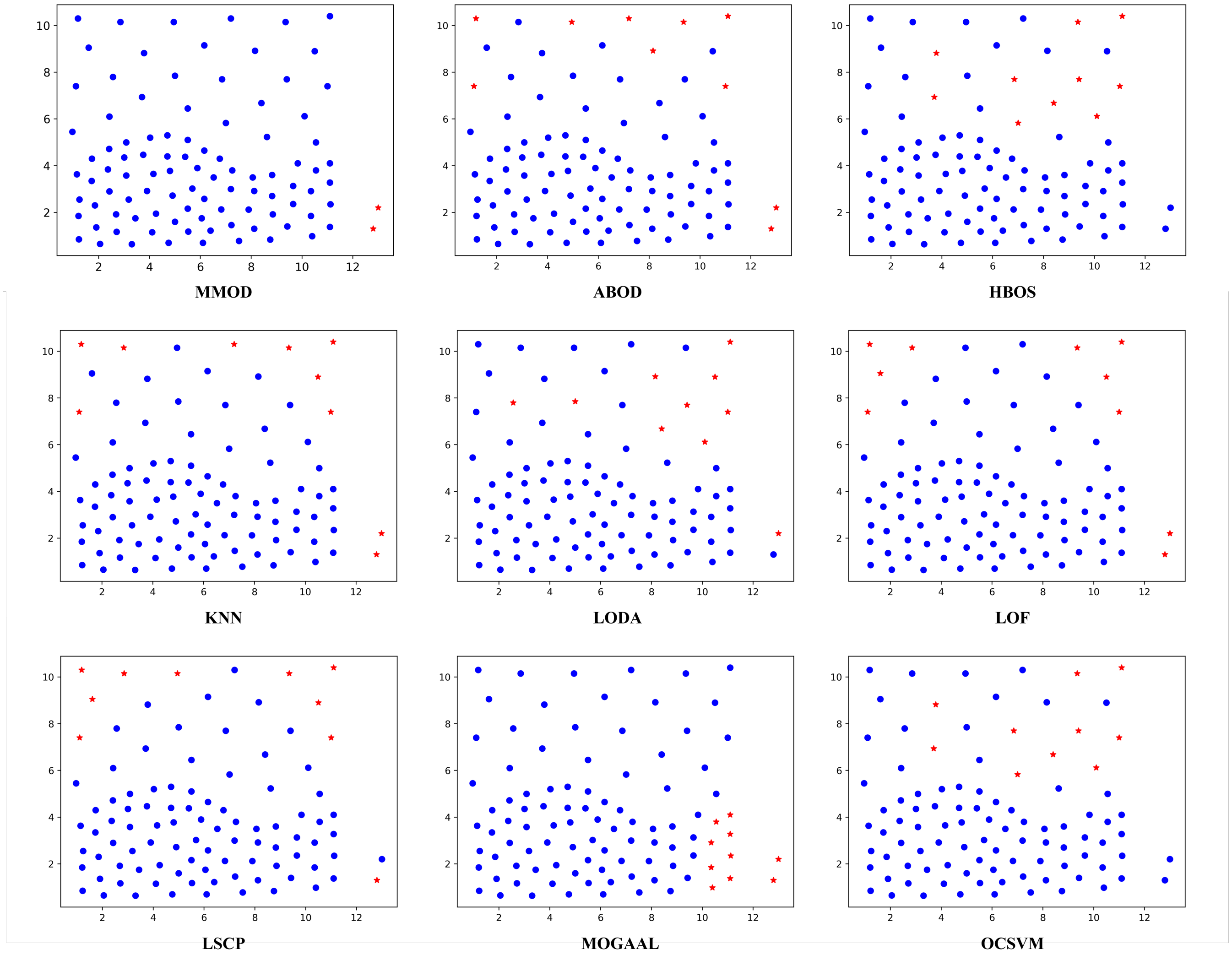

Figure B. Nine algorithms' outlier detection results on the synthetic "Two densities" dataset. Detected outliers are in red.

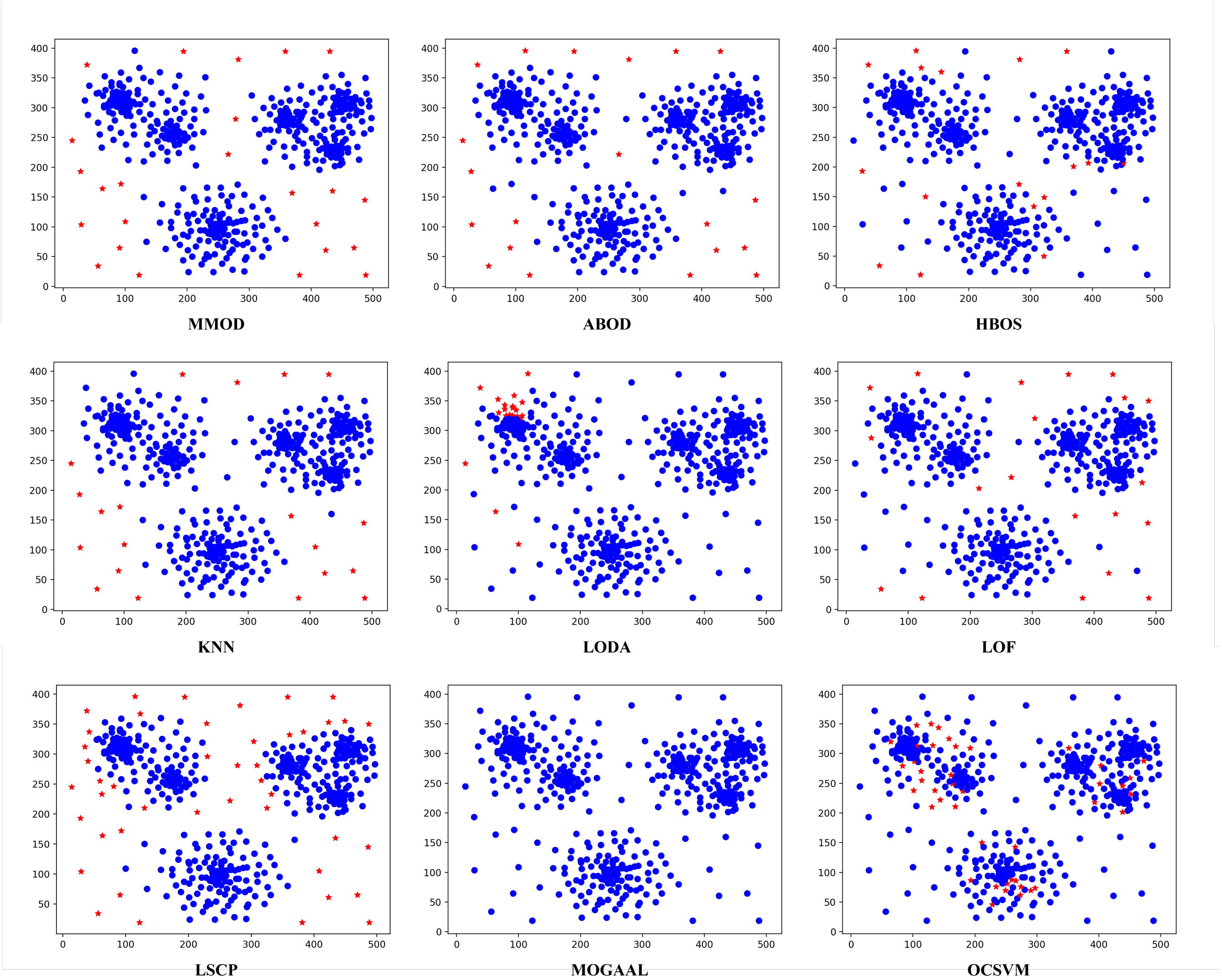

Figure C. Nine algorithms' outlier detection results on the synthetic "Three clusters" dataset. Detected outliers are in red.

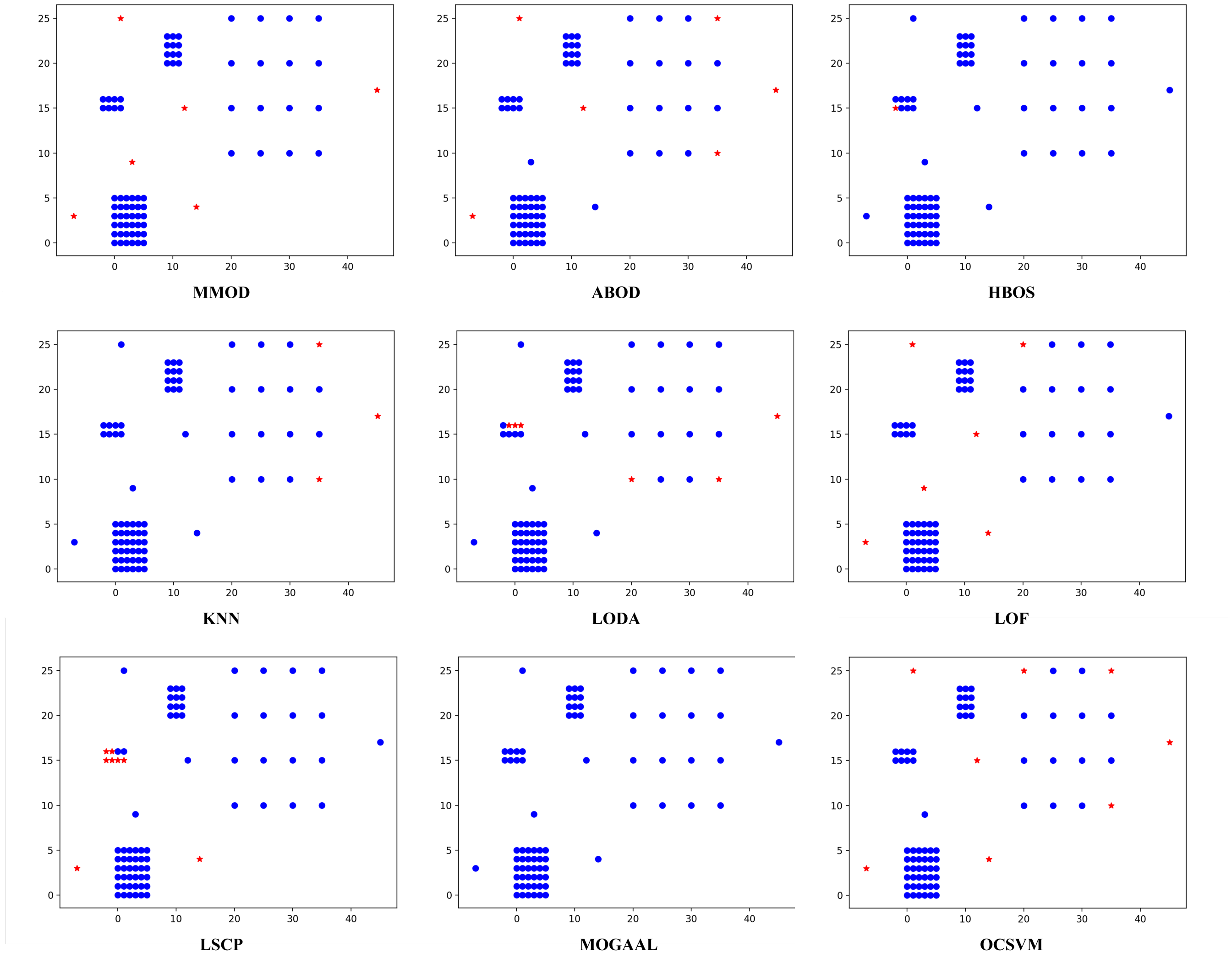

Figure D. Nine algorithms' outlier detection results on the synthetic "Four densities" dataset. Detected outliers are in red.

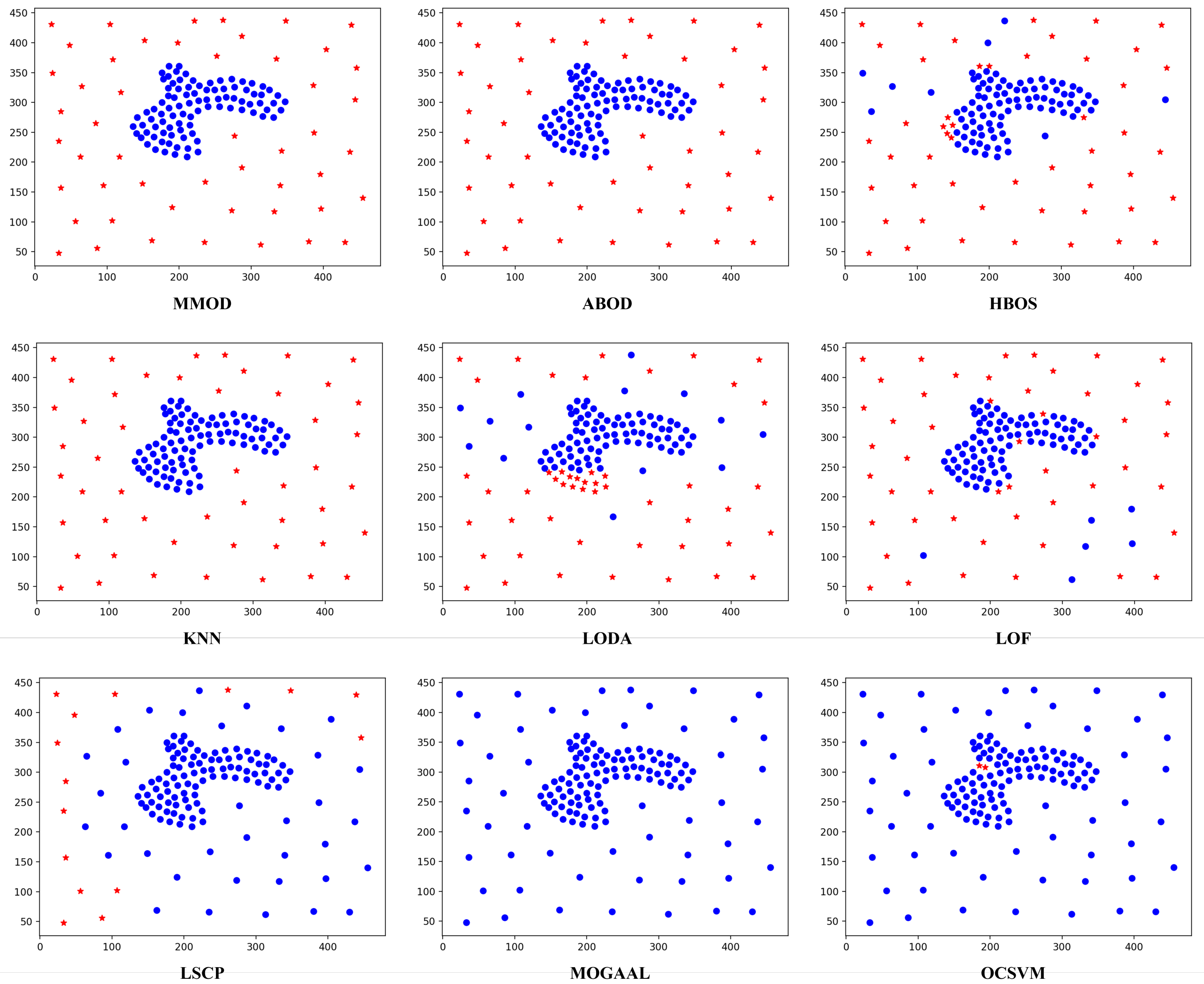

Figure E. Nine algorithms' outlier detection results on the synthetic "Uniform outliers" dataset. Detected outliers are in red.

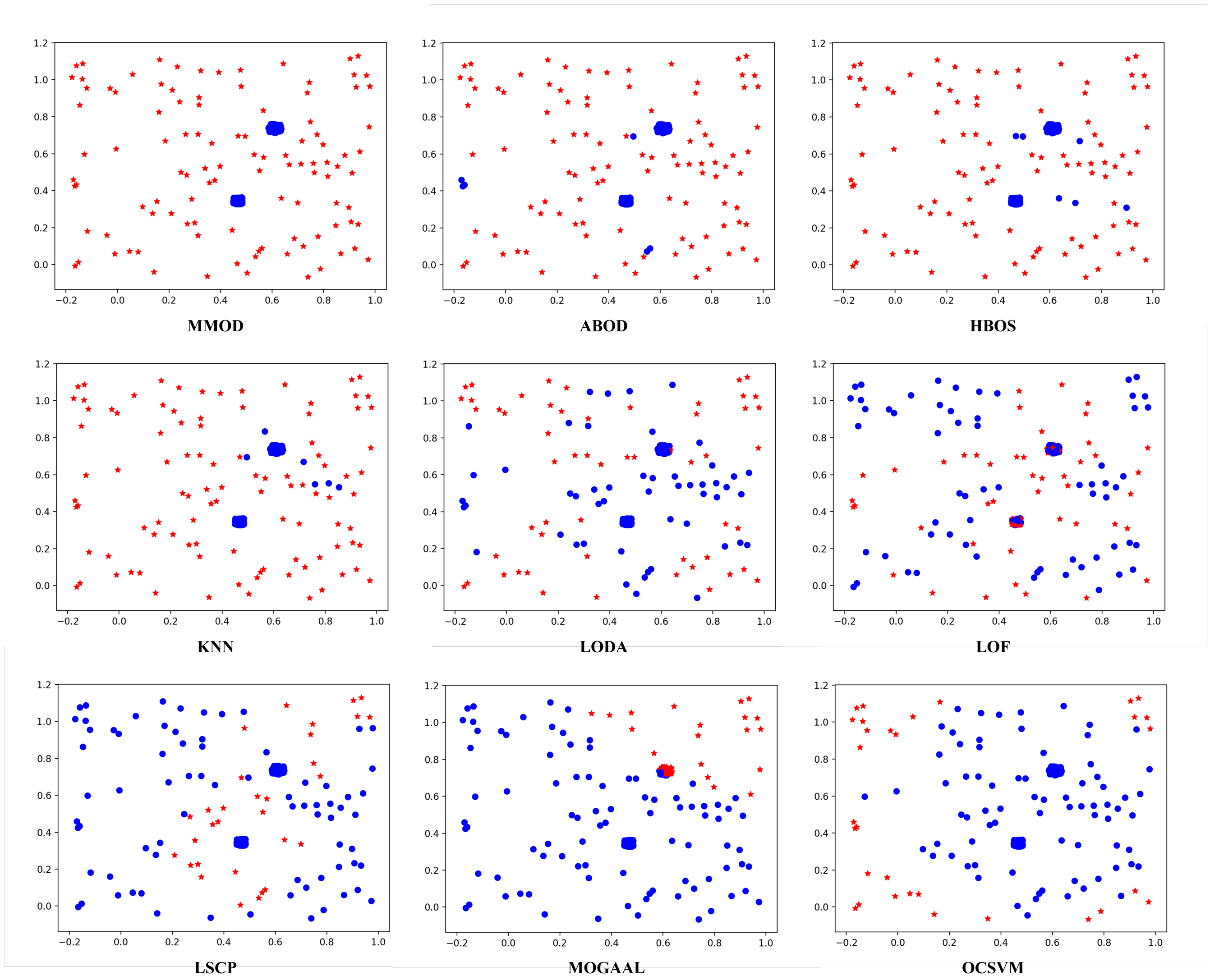

Figure F. Nine algorithms' outlier detection results on the synthetic "Arbitrary outliers" dataset. Detected outliers are in red.
